# Dark matters: Contrasting responses of stream biofilm to browning and loss of riparian shading

**DOI:** 10.1111/gcb.16279

**Published:** 2022-06-15

**Authors:** Jussi Jyväsjärvi, Maria Rajakallio, Joanna Brüsecke, Kaisa‐Leena Huttunen, Ari Huusko, Timo Muotka, Sami J. Taipale

**Affiliations:** ^1^ Ecology and Genetics Research Unit University of Oulu Oulu Finland; ^2^ Natural Resources Institute Finland (Luke) Paltamo Finland; ^3^ Department of Biological and Environmental Science University of Jyväskylä Jyväskylä Finland

**Keywords:** DOC, fatty acids, forestry, mesocosm experiment, multiple stressors, periphyton

## Abstract

Concentrations of terrestrial‐derived dissolved organic carbon (DOC) in freshwater ecosystems have increased consistently, causing freshwater browning. The mechanisms behind browning are complex, but in forestry‐intensive regions browning is accelerated by land drainage. Forestry actions in streamside riparian forests alter canopy shading, which together with browning is expected to exert a complex and largely unpredictable control over key ecosystem functions. We conducted a stream mesocosm experiment with three levels of browning (ambient vs. moderate vs. high, with 2.7 and 5.5‐fold increase, respectively, in absorbance) crossed with two levels of riparian shading (70% light reduction vs. open canopy) to explore the individual and combined effects of browning and loss of shading on the quantity (algal biomass) and nutritional quality (polyunsaturated fatty acid and sterol content) of the periphytic biofilm. We also conducted a field survey of differently colored (4.7 to 26.2 mg DOC L^−1^) streams to provide a ‘reality check’ for our experimental findings. Browning reduced greatly the algal biomass, suppressed the availability of essential polyunsaturated fatty acids, especially eicosapentaenoic acid (EPA), and sterols, but increased the availability of terrestrial‐derived long‐chain saturated fatty acids (LSAFA). In contrast, loss of shading increased primary productivity, which resulted in elevated sterol and EPA contents of the biofilm. The field survey largely repeated the same pattern: biofilm nutritional quality decreased significantly with increasing DOC, as indicated particularly by a decrease of the *ω*‐3:*ω*‐6 ratio and increase in LSAFA content. Algal biomass, in contrast, was mainly controlled by dissolved inorganic nitrogen (DIN) concentration, while DOC concentration was of minor importance. The ongoing browning process is inducing a dramatic reduction in the nutritional quality of the stream biofilm. Such degradation of the major high‐quality food source available for stream consumers may reduce the trophic transfer efficiency in stream ecosystems, potentially extending across the stream‐forest ecotone.

## Graphical abstract

We conducted a mesocosm experiment to explore the effects of browning and shading loss on the quantity and quality of the stream biofilm. The experiment was supplemented with a field survey of differently colored streams to provide a ‘reality check’ for our experimental findings. Browning reduced algal biomass, suppressed the availability of eicosapentaenoic acid (EPA), and sterols, but increased the availability of terrestrial‐derived long‐chain saturated fatty acids (LSAFA). The field data repeated the same pattern: biofilm nutritional quality decreased significantly along with browning. The ongoing browning process is inducing a dramatic reduction in the nutritional quality of the stream biofilm.

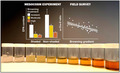

## 1 INTRODUCTION

Concentrations of terrestrial‐derived dissolved organic carbon (t‐DOC) in boreal lakes and rivers have increased consistently over the past few decades, causing a phenomenon known as ‘brownification’ or ‘browning’ (de Wit et al., 2016; Monteith et al., 2007). Many hypotheses have been put forward to explain this phenomenon, including land use intensification, reduced acid deposition and increased temperature (Larsen et al., 2011; Solomon et al., 2015). Currently, the best‐supported hypothesis is that the decrease in atmospheric sulfate deposition and associated recovery from acidification have resulted in increased soil pH, with a subsequent increase in DOC solubility (Monteith et al., 2007). While the mechanisms behind browning are complex and much debated, its repercussions on ecosystem services and human health are better defined (Kritzberg et al., 2020; Taipale, Vuorio, et al., 2016). Browning has also important ecological consequences, mainly via its effects on water transparency (Solomon et al., 2015). DOC in lakes constrains primary production via light absorption but it also binds nutrients, thus initially stimulating primary production (Fork, Karlsson, & Sponseller, 2020). This effect is strongest at low DOC concentrations, often resulting in a hump‐shaped relationship between t‐DOC and gross primary productivity (Deininger et al., 2017; Olson et al., 2020). While most lentic studies have focused on pelagial habitats, Fork, Karlsson, and Sponseller (2020) recently demonstrated a corresponding unimodal response of benthic algal production to increasing DOC concentrations in Swedish lakes.

The role of t‐DOC has been extensively studied in lake ecosystems while its importance to primary production and food webs in streams has received less attention (but see Burrows et al., 2021; Kuglerová et al., 2021), despite a recent study showing that browning may be particularly strong in forested headwater streams (Fork, Sponseller, & Laudon, 2020). Especially peatland drainage, practiced to enhance forest growth on moist soils, has been detected to be a major factor causing browning of boreal streams (Asmala et al., 2019; Nieminen et al., 2021). Similarly, harvesting close to the stream edge may increase DOC input and thus contribute to stream browning (Kuglerová et al., 2021).

A conventional means of reducing the adverse impacts of forestry on recipient freshwater ecosystems is to retain an intact strip of riparian vegetation between the stream and the harvested area. A recent survey of riparian buffer management in three major forestry jurisdictions (British Columbia Canada, Finland, Sweden) revealed, however, that a substantial proportion of small forest streams are left with thin or no riparian buffers (Kuglerová et al., 2020). The lack of adequate buffers reduces canopy shading, causing increased light intensity and higher stream water temperature. Under such conditions, the biomass and productivity of periphytic algae, the key group of primary producers in streams, usually increase (Jyväsjärvi et al., 2020; Kiffney et al., 2003). At the same time, however, the quality of biofilm as food for higher trophic levels may decrease because increased illumination may alter algal community composition, changing the availability of *ω*‐3 and *ω*‐6 polyunsaturated fatty acids (PUFAs) and sterols (Arts et al., 2009; Napolitano, 1994; Taipale, Vuorio, et al., 2016).

Periphytic algal communities in near‐pristine streams are typically dominated by diatoms which contain eicosapentaenoic acid (EPA; 20:5*ω*‐3), a long chain PUFA physiologically essential for aquatic invertebrate consumers (Parrish, 2009; Twining et al., 2015). Periphytic algal communities are highly responsive to nutrient enrichment and illumination (Burrows et al., 2021), both of which typically enhance dominance by cyanobacteria which may contain high amounts of alpha‐linolenic acid (ALA; 18:3*ω‐*3), but no EPA or DHA (docosahexaenoic acid; 22:6*ω*‐3; Taipale, Hiltunen, et al., 2016; Senar et al., 2019). Furthermore, cyanobacteria do not contain any sterols, resulting in sterol limitation in cyanobacteria‐dominated ecosystems (Martin‐Creuzburg et al., 2008; Peltomaa et al., 2017). Similarly, freshwater green algae can contain high amounts of ALA, stearidonic acid (SDA; 18:4*ω*‐3) and some sterols, but are poor in EPA (Taipale, Hiltunen, et al., 2016). EPA and DHA may be bioconverted from short‐chain PUFA, but with a generally low efficiency (see Brett & Müller‐Navarra, 1997), which is why many aquatic consumers must obtain EPA or DHA directly from their diet. Heterotrophic bacteria can also be abundant in stream biofilm, but they do not contain any PUFA or sterols, providing therefore insufficient diet for invertebrates (Taipale et al., 2012). Similarly, terrestrial‐derived organic matter may contain a substantial amount of carbohydrates but are poor in sterols and PUFA (Taipale, Galloway, et al., 2016; Torres‐Ruiz & Wehr, 2020). The importance of terrestrial‐derived particulate organic matter (t‐POM) in lentic pelagic food webs has been debated for the last two decades but corresponding studies in streams are few (but see Guo et al., 2015; Torres‐Ruiz et al., 2007; Twining et al., 2017). It has been suggested, however, that high loadings of terrestrial material may impair the nutritional value of basal resources for freshwater consumers (Creed et al., 2018).

Browning alters the light climate, suppressing productivity of periphytic algae (Vasconcelos et al., 2016). Conversely, reduction of riparian shading increases irradiance and thereby stimulates algal productivity (Jyväsjärvi et al., 2020), thus potentially counteracting the effect of browning (antagonistic response) on algal biomass. Effects of browning and light on periphyton quality are less obvious and harder to predict. However, low light in highly shaded streams has been reported to increase PUFA production whereas open canopy stimulates the synthesis of saturated fatty acids (Guschina & Harwood, 2006; Guo, Kainz, Sheldon, & Bunn, 2016). Impacts of browning on primary producers and their fatty acids have not been previously addressed in streams, and lake studies have yielded variable outcomes, with both positive (Gutseit et al., 2007), neutral (Strandberg et al., 2020), and negative (Senar et al., 2019) responses of lake seston PUFA content to browning, suggesting that effects of browning on phytoplankton communities may differ among geographical regions and environmental contexts (Strandberg et al., 2020). Stream periphyton is usually dominated by diatoms, chlorophytes (green algae) and cyanobacteria (Allan & Castillo, 2007) whereas many taxonomic groups that have important roles in lake food webs are lacking from, or occur sporadically, in streams. It may therefore be that lake studies do not provide much theoretical support for predicting the impacts of browning on stream periphyton biomass and fatty acid profiles.

We conducted a stream mesocosm experiment to explore the individual and combined effects of two light‐related stressors, that is, browning and loss of riparian shading, on the quantity and quality of the periphytic biofilm. We expected (1) increasing browning to reduce algal productivity (lower chlorophyll *a* accrual rate) whereas (2) loss of shading should have an opposite effect, compensating the loss of algal biomass caused by browning. For qualitative changes, we expected (3) browning to suppress the growth of diatoms, and thus constrain EPA synthesis (Taipale, Hiltunen, et al., 2016). We also expected (4) phytosterols synthesized by algae to respond similarly to browning, thus enhancing the reduction of biofilm quality for stream consumers under browning. We further expected (5) excess irradiance caused by the loss of riparian shading to reduce PUFA production, thus potentially resulting in a synergistic combined effect of browning and loss of shading on stream biofilm quality. Finally, we assessed the realism of our experimental findings by conducting a field survey where we quantified the algal biomass and fatty acid composition of benthic biofilm in 45 streams in central Finland covering a wide DOC gradient from <5 to 26 mg L^−1^.

## 2 MATERIALS AND METHODS

### 2.1 Experimental setup

We manipulated water absorbance and DOC content (i.e. browning) and shade in flow‐through artificial stream channels at the Natural Resources Institute Finland's research station (Kainuu Fisheries Research Station [N 64° 24.240' E 27° 31.320']), Paltamo, central Finland. Water from the nearby Lake Kivesjärvi is pumped into a 300 m^2^ pond, which enters a 30‐m long, permanently flowing stream before draining into 24 m × 1.5 m experimental channels (Figure S1). Incoming water is nutrient poor (PO_4_: 3.3 ± SE 0.9 μg L^−1^; NO_3_ + NO_2_: 19.3 ± 0.7 μg L^−1^), neutral (pH: 7.0 ± 0.04), and mesohumic (DOC: 9.8 ± 0.1 mg L^−1^).

**FIGURE 1:**
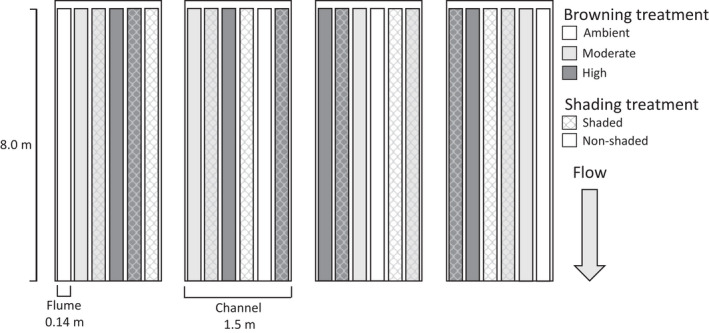
A schematic presentation of the experimental design showing the random assignment of browning and shading removal treatments within each channel (block), replicated across four channels.

The lowermost section of four channels was divided into six parallel 8 × 0.14 m flumes made of horizontally split plastic drainpipes (Figure S1). This resulted in 24 experimental units, six in each of the four main channels in a randomized order (Figure 1). Our experiment thus used a fully randomized block design with four replicates for each treatment combination. We manipulated water absorbance/DOC at three levels (ambient vs. moderate vs. high) and shading at two levels (shade vs. no shade).

Prior to the experiment, medium‐sized gravel (Ø 2–5 cm) was washed and added to the bottom of each flume. Water supply to the channels was controlled through a valve and the flow was adjusted at 0.36 (±SD 0.05) L s^−1^ in each flume. The average (±SD) water depth in the flumes was 7 (±0.6) cm. The experiment ran for 43 days, from 9th of August to 19th of September 2018.

### 2.2 Shading treatment

We used Ecoplant–garden fabric (Thujor.se) to mimic riparian canopy shading. The fabric reduced incoming light by 70% (Figure S2a), closely equaling the shading provided by undisturbed riparian forests (Jyväsjärvi et al., 2020). Shading removal did not alter water temperatures (Figure S2b).

### 2.3 Browning treatment

We used a commercially available product (Huminfeed WGS [hereafter HF], Humin‐tech GmbH, Düsseldorf, Germany) to manipulate absorbance and DOC content of the stream water. HF is obtained through alkaline extraction of highly oxidised German lignite, and it has been widely applied in ecological studies to mimic different browning scenarios (Lebret et al., 2018; Nydahl et al., 2019; Urrutia‐Cordero et al., 2016; Wilken et al., 2018). The use of lignite‐derived browning agents, such as HF, have been recently questioned, due primarily to increased particle formation and light‐induced loss of the browning effect (Scharnweber et al., 2021). As our experiment was conducted in artificial streams and with continuous supply of HF, we consider these confounding effects to be minor in our experiment. Importantly, largely similar patterns of browning impacts on biofilm quality were echoed in our field observations, suggesting that our results were not experimental artifacts caused by HF but represented a real‐world situation (see Results).

The HF powder was dissolved at estimated doses (moderate and high concentration, see below) into two 2000 L head tanks filled with filtered (Ø 500 μm) stream water. The tanks were filled with new HF solution at 24‐h intervals. The solution was continuously supplied from the head tanks into the upstream end of each target flume using Watson Marlow 504S peristaltic pumps (Watson Marlow Bredel, Wilmington, MA, USA). Based on a pilot experiment, we set the HF solution pumping rate for each flume at 40 ml min^−1^, which resulted in a 2.7‐fold increase in water absorbance (436 nm) in moderate and a 5.5‐fold increase in high‐browning treatments (Figure S3). This yielded an increase of DOC concentrations by factors of 1.4 and 1.9, respectively (ambient 9.7 mg L^−1^ (SE ± 0.1); moderate 13.2 mg L^−1^ (±0.3); high 16.8 mg L^−1^ (±1.1)). The ambient water colour during the experiment was on average 72 mg Pt L^−1^ (SE ± 4.9) and it was increased to 242 (±12.0) and 436 (±35.8) mg Pt L^−1^ in moderate and high‐browning treatments, respectively. These values correspond to 20%, 75% and 98% quantiles of water colour monitoring data for streams in Finland during 2010–2017 (Finnish Environment Institute, HERTTA database; Figure S4). We monitored water absorbance spectrophotometrically (Genesys™ 10S UV–Vis, Thermo Scientific, USA) daily in each flume to verify that treatment levels remained stable and did not overlap during the experiment (Figure S3). DOC concentrations were monitored from filtered (Ø 0.45 μm Whatman GF/F) water samples three times during the experiment by infrared spectrometry with Shimadzu TOC‐V_CPH_ analyzer (Shimadzu Scientific Instruments, Kyoto, Japan). As the HF contains various other elements in addition to carbon (see Meinelt et al., 2007), our moderate and high browning treatments resulted in a subtle increase of NO_3_ + NO_2_ (22.9 and 25.4 μg L^−1^ in moderate and high browning treatments, respectively) and PO_4_ (5.4 and 6.2 μg L^−1^, respectively) concentrations compared to ambient conditions (see above). This was desirable as the main goal of using HF to mimic water browning was to increase DOC and absorbance while maintaining nutrients almost unaltered. Furthermore, such a slight increase in nutrients is an intrinsic component of the browning process (e.g. Deininger et al., 2017). Moderate and high browning treatment reduced light intensities by 15% and 28% in shaded flumes and by 11% and 27% in non‐shaded flumes, respectively (Figure S2a). Browning treatments had no effect on water temperatures (Figure S2b).

### 2.4 Other physico‐chemical measurements

Water temperature and light intensity were monitored at 30‐min intervals in one flume for each treatment, using data loggers (HOBO Pendant, Onset, Massachusetts) attached to a rebar at the end of a flume. Water samples for electrical conductivity (mS m^−1^), pH, PO_4_ (μg L^−1^) and NO_3_ + NO_2_ (μg L^−1^) were taken three times during the experiment and the samples were analyzed using national standards (National Board of Waters, 1981).

### 2.5 Biofilm sampling

The upstream end (1 m) of each flume was reserved as a mixing zone for HF and the remaining 7 m was used as the experimental arena where we inserted six 10 × 10 cm unglazed ceramic tiles to serve as substrate for stream biofilm. For quantification of algal biomass (chlorophyll *a*), three tiles in each flume were stratified into three equidistant (2.3 m) longitudinal flume sections and the tiles were sampled at the end (week 6) of the experiment. The remaining three similarly distributed tiles were used for fatty acid and sterol analyses, and they were also sampled at the end of the experiment. We did not attempt to exclude algae‐feeding benthic invertebrates (grazers) from the tiles.

The upper surfaces of the chlorophyll *a* tiles were brushed into a small amount (100 ml) of filtered stream water using a toothbrush and the material was immediately filtered onto 0.7 μm Whatman GF/F glass fiber filter, which was then wrapped in aluminum foil and frozen (−20°C). Algal pigments were extracted with 90% ethanol and chlorophyll *a* concentration was measured spectrophotometrically (Shimadzu UV‐1601PC) using fluorescence readings at 665 and 750 nm. The biofilm of the other three tiles (sterol and fatty acid analyses) was scraped off with a sterile razor blade into 50 ml plastic vials, which were immediately frozen and kept at −20°C. Upon arrival to the laboratory, fatty acid and sterol samples were transferred to −80°C.

### 2.6 Fatty acid analyses

Prior to fatty acid and sterol analyses, samples were freeze‐dried for 48 h and homogenized. The three replicate samples from the same flume were pooled before analyses to guarantee a sufficient amount of sample material. Lipids were extracted from the pooled samples (16–20 mg DW) using the Folch method (Folch et al., 1957): chloroform: methanol 2:1 mixture was sonicated for 10 min, after which 0.75 mL of distilled water was added. Fatty acids of total fraction were methylated in acidic conditions. Toluene and sulfuric acid were used for the transesterification of fatty acid methyl esters (FAMEs) at 50°C for 16 h which is an optimal method for methylation of PUFA. FAMEs were analyzed with gas chromatograph (Shimadzu Ultra, Kyoto, Japan) equipped with a mass detector (GC–MS), using helium as a carrier gas (linear velocity = 36.3 cm s^−1^). Temperature of the injector was 270°C and we used a splitless injection mode (for 1 min). Temperatures of the interface and ion source were 250°C and 220°C, respectively. Phenomenex® (Torrance, California, USA.) ZB‐FAME column (30 m × 0.25 mm × 0.20 μm) with 5 m Guardian was used with the following temperature program: 50 °C was maintained for 1 min, then the temperature was increased at 10°C min^−1^ to 130°C, followed by 7°C min^−1^ to 180°C, and 2°C min^−1^ to 200°C, then held at that temperature for 3 min, and finally heated at 10°C min^−1^ to 260°C. Total program time was 35.14 min and solvent cut time 9 min. Fatty acids were identified by the retention times (RT) using specific ions (Taipale, Galloway, et al., 2016) which were also used for quantification. Fatty acid concentrations were calculated using calibration curves based on known standard solutions (15, 50, 100 and 250) of a FAME standard mixture (GLC standard mixture 566c, Nu‐Chek Prep, Elysian, Minnesota, U.S.A.) and using recovery percentage of internal standards. The Pearson correlation coefficient was >0.99 for each individual fatty acid calibration curve. Additionally, we used 1,2‐dinonadecanoyl‐sn‐glycero‐3‐ phosphatidylcholine (Larodan, Malmö, Sweden) and free fatty acid of C23:0 (Larodan, Malmö, Sweden) as internal standards and for calculation of the recovery percentages.

### 2.7 Sterol analysis

The extracted lipids were purified using Bond Elut LRC‐SI cartridges (500 mg). The resin of the cartridge was activated using the mixture of chloroform and methanol (1:1). Subsequently, total lipids were dissolved to the 300 μl of chloroform and applied to the cartridge. Chloroform (10 ml) was used to elute neutral lipids (sterols). After the evaporation neutral lipid fraction was diluted in 100 μl of pyridine and silylated with 70 μl of N,O‐bis[trimethylsilyl trifluoroacetamide] (BSTFA), trimethylchlorosilane (TMCS) at 70°C. Trimethylsilyl (TMS) derivatives of sterols were analyzed with GC–MS (Shimadzu) equipped with an Phenomenex ZB‐5 column (30 m 9 0.25 mm 9 0.15 lm) using the following temperature program: 150°C for 1 min, then raised at a rate of 15°C min^−1^ to 280°C, followed by 2°C min^−1^ to 320°C, and finally held for 10 min. Helium was used as a carrier gas with an average velocity of 31 cm s^−1^. Sterols were quantified using authentic standards from Sigma‐Aldrich and Larodan and 5‐a‐cholestan as an internal standard. Sterols were identified using characteristic ions (Taipale, Galloway, et al., 2016).

### 2.8 Field survey

Unglazed ceramic tiles (10 × 10 cm) were attached to house bricks (*n* = 8 tiles at each site) and were incubated in the stream water for 5 weeks in early August to September 2019 in 45 1st‐to‐2nd order tributaries of River Iijoki in central Finland (Figure S5). The study streams ranged from non‐humic, clear water (4.7 mg DOC L^−1^) to strongly brownified (26.2 mg DOC L^−1^) and varied also in other environmental attributes, such as nutrient concentrations, pH and catchment and stream size (see Table S1). Water chemistry variables were measured in August 2019 using the standard methods described above and water temperature was measured during August–September with submerged HOBO Pendant loggers at 30‐min intervals. Current velocity (cm s^−1^; Schiltknecht® MiniAir20) and water depth (cm) were measured at three positions along five randomly placed transects perpendicular to the flow. Stream width (m) was measured at the same transects. Mean riparian canopy cover (%) was estimated from ten fisheye‐lens upward photographs using the GLAMA mobile phone application (Tichý, 2016). At the end of the incubation period, periphyton accumulated on tiles (four tiles per site) was scraped off with a toothbrush and the samples were analysed for chlorophyll *a* biomass as described above. Biofilm for the fatty acid analyses was sampled from another set of four tiles using a premoistened sterile Speci‐Sponge™ (Nasco, Modesto, California). This sampling technique has been frequently used in stream biofilm studies (e.g. Lear et al., 2009). To guarantee that enough material was obtained, samples were pooled across the four tiles at a site. The sponges were transported to the laboratory, stored at −80 °C and subsequently freeze‐dried for 48 h. Fatty acids were analyzed as described above.

### 2.9 Data analyses

Prior to statistical analyses, fatty acids were grouped by their chemical structure (see Taipale et al., 2013) in four groups: (1) *ω*‐3 polyunsaturated fatty acids, (2) *ω*‐6 polyunsaturated fatty acids, (3) long‐chain saturated fatty acids of mainly terrestrial origin (LSAFA), and (4) bacterial fatty acids (BFA) (Table S2). Three of these (1), (3), and (4) were used as response variables in data analyses. Eicosapentaenoic acid (EPA; 20:5 *ω*‐3) content and the ratio of *ω*‐3 and *ω*‐6 fatty acids were also used as response variables to assess the nutritional quality changes in stream biofilm.

For the experimental data, we constructed linear mixed effect models (LMM) to identify the effects of each treatment and their interactions on univariate responses (algal biomass [mg Chl *a* m^−2^]); contents (μg mg C^−1^) of each fatty acid group (see above); *ω*‐3/*ω*‐6 ratio and total sterol content (ng mg C^−1^). We fitted flumes nested within main channels as random effects to account for background variation among replicate flumes. Algal biomass was measured in multiple (n = 3) sections per flume, and we therefore specified section identity also as a random effect nested within flumes. The shading and browning treatments were treated as fixed effects. Likelihood ratio tests and comparison of AIC (Akaike's information criterion) values were used to compare models with and without the interaction term (browning × shading) and the random effects in the final models were estimated using restricted maximum likelihood estimation (REML). Each model was validated by examining residual plots and, in cases of heteroscedastic variances, we included *varIdent* variance function (Pinheiro et al., 2020) to allow for heterogeneity in variance among different browning levels. In case of significant treatment interactions, we evaluated significance of simple effects (i.e. browning effect on each level of shading) using *emmeans* package (Lenth, 2020) implemented in R statistical software (R Core Team, 2020). LMMs were run using *nmle* (Pinheiro et al., 2020) and *lmerTest* (Kuznetsova et al., 2017) R packages.

The relative content data of individual fatty acids and sterols out of total fatty acids/sterols were visualized using non‐metric multidimensional scaling (NMDS) and *envfit*‐function of the *vegan* R‐package (Oksanen et al., 2019) was applied to superimpose the fatty acid groups and total sterols onto the ordination plot. Contribution of each experimental treatment to fatty acid and sterol composition was examined by permutational (*n* = 9999) multivariate analyses of variance (PERMANOVA), using the *adonis*‐function of the *vegan* R package (Oksanen et al., 2019) based on Bray–Curtis dissimilarities on relative content data. Shading, browning and their interactions were included in the PERMANOVA models to evaluate their relative contributions (and associated statistical significance) to total variance of fatty acid and sterol composition. In case of a significant interaction, we performed separate PERMANOVAs for shaded and non‐shaded flumes to test for compositional differences among the browning treatments (ambient vs. moderate/high).

The field survey data were analyzed with ordinary least squares regressions using algal biomass (Chl‐*a*, mg m^−2^), contents (%) of LSAFA, *ω*‐3, and EPA fatty acids, and the *ω*‐3/*ω*‐6 ratio as the response variables and the measured environmental attributes (Table S1) as the candidate explanatory variables. The most parsimonious regression model for each response variable was selected using the ‘all‐possible‐subsets’ approach (*regsubsets*‐function in the *leaps* R package [Lumley, 2020]). The model with the lowest Bayesian information criterion (BIC; Neath & Cavanaugh, 2012) was considered as the most parsimonious one. All predictor variables (except pH) were log_10−_transformed to improve the normality of model residuals.

## 3 RESULTS

### 3.1 Algal biomass and quantities of fatty acids and sterols

**FIGURE 2:**
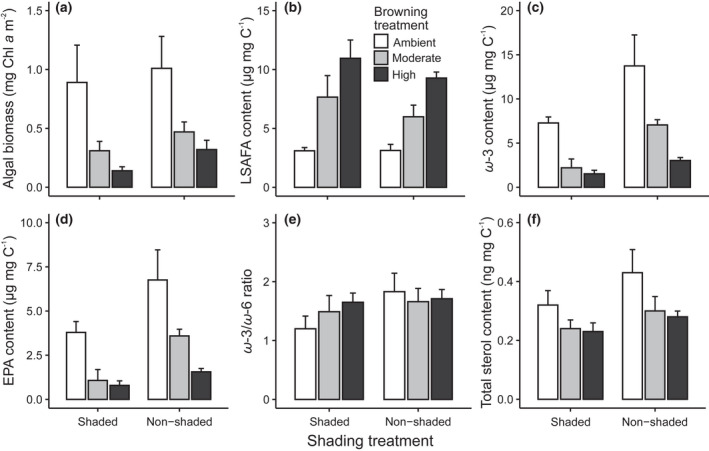
Averages (±95% CI) of (a) algal biomass (chlorophyll a), content of (b) long‐chain saturated fatty acids (LSAFA), (c) ω‐3 polyunsaturated fatty acids, (d) eicosapentaenoic acid (EPA; 20:5ω‐3), (e) the ratio of ω‐3 and ω‐6 polyunsaturated fatty acids, and (f) total sterol content in different experimental treatments.

Control (ambient DOC–shaded) chlorophyll *a* content averaged 0.89 (SE ± 0.16) mg m^−2^ after six‐week incubations. Moderate browning reduced chlorophyll *a* content by on average 65.2% (standardized *β* = −.56, *t* = −5.09, *p* < .001) and high browning by 83.1% (*β* = −.72, *t* = −6.59, *p* < .001; Figure 2a). Loss of shading resulted in algal biomass increase by on average 18.4% (*β* = .15, *t* = 3.55, *p* = .002; Figure 2a). The interaction term was non‐significant (*p* = .95).

The content of long‐chain saturated fatty acids (LSAFA) increased in both moderate (*β* = 3.71, *t* = 6.41, *p* < .001) and high browning (*β* = 7.00, *t* = 12.10, *p* < .001) treatments, while the loss of shading had a weaker negative effect on LSAFA (*β* = −1.10, *t* = −2.34, *p* = .031; Figure 2b). The *ω*‐3 polyunsaturated fatty acids were strongly reduced by both moderate (*β* = −5.07, *t* = −3.74, *p* = .002) and high (*β* = −5.76, *t* = −4.42, *p* < .001) browning, while the loss of shading increased (*β* = 6.46, *t* = 3.54, *p* = .003) their content (Figure 2c).

The among‐treatment differences in *ω‐*3 fatty acids reflected mainly variation in EPA, which indicated the overall browning‐induced reduction of EPA‐rich diatoms. Similar to *ω*‐3, biofilm EPA content was markedly reduced by both moderate (*β* = −2.71, *t* = −4.64, *p* < .001) and high (*β* = −2.99, *t* = −5.13, *p* < .001) browning, while the loss of shading increased (*β* = 2.98, *t* = 5.10, *p* < .001) their content (Figure 2d). The significant interaction term indicated that, in shaded conditions, the EPA content in both moderate and high browning treatments was strongly reduced (both *p* < .005) compared to control, but the difference between moderate and high browning was non‐significant (*t* = .49, *p* = .879; Figure 2d). In non‐shaded conditions, all differences among the browning treatments were significant (all *p* < .009; Figure 2d). The ratio of *ω*‐3 and *ω*‐6 fatty acids was weakly increased by moderate (*β* = .29, *t* = 2.45, *p* = .07) and high (*β* = .45, *t* = 3.85, *p* = .004) browning, but only in shaded conditions (interaction *p* = .009) (Figure 2e), reflecting the parallel reduction of both omega fatty acids with increased browning (see Figure S6a). Shading removal elevated the *ω*‐3/*ω*‐6 ratio (*β* = .63, *t* = 5.35, *p* < .001; Figure 2e).

Similar to fatty acids (exclusive of LSAFA), total sterol content was reduced in moderate (*β* = −.10, *t* = −3.81, *p* = .001) and high browning (*β* = −.12, *t* = −4.69, *p* < .001) treatments, whereas loss of shading had a positive effect on sterols (*β* = .05, *t* = 6.24, *p* < .001), with no interaction between the two treatments (Figure 2f). Bacterial fatty acid (BFA) content was overall very low (average 0.74 μg mg C^−1^) and reduced by moderate (*β* = −.55, *t* = −7.14, *p* < .001) and high browning (*β* = −.74, *t* = −9.55, *p* < .001), whereas shading had no effect on BFA (*β* = .05, *t* = .81, *p* > .05) (Figure S6b).

### 3.2 Biofilm fatty acid and sterol composition

**FIGURE 3:**
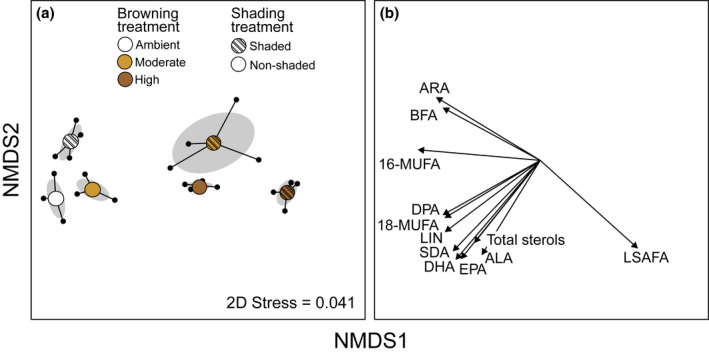
NMDS ordination of the biofilm fatty acid composition, showing separation of the experimental treatments in the ordination space (a) and fits (i.e. arrow lengths) of the ordination axes and fatty acid groups (b). Light‐grey ellipses in the left‐hand panel depict 95% confidence ellipses around treatment centroids.

Browning, loss of shading and their interaction had significant effects on biofilm fatty acid composition, explaining 73%, 13%, and 6% of total variance, respectively (Table 1). In shaded flumes, fatty acid composition differed strongly from ambient levels in both moderate (PERMANOVA: *F*_1,9_ = 41.76, *p* < .001) and high (*F*_1,9_ = 66.08, *p* = .002) browning treatments (Figure 3a). In non‐shaded flumes, the compositional difference between ambient and moderate browning was smaller (*F*_1,9_ = 8.83, *p* = .01), whereas ambient and high browning differed significantly [*F*_1,9_ = 70.35, *p* < .001; Figure 3a]. In the NMDS ordination space, the shaded, low DOC conditions were characterized by bacterial fatty acids and the polyunsaturated *ω*‐6 fatty acid, arachidonic acid (ARA; 20:4*ω*‐6), whereas the shaded, high‐DOC flumes were dominated by LSAFAs (Figure 3b). The *ω‐*3 fatty acids (EPA and DHA) and total sterols increased towards moderate‐to‐ambient DOC, non‐shaded flumes (Figure 3b).

**TABLE 1:** Summary of PERMANOVA for the effects of browning, shading removal and their interaction on biofilm fatty acid composition

	*Df*	*SS*	*MS*	*R* ^*2*^	*F value*	*p*
Browning	2	2198.4	1099.2	.73	89.0	<.001
Shading	1	393.0	393.0	.13	31.8	<.001
Browning × Shading	2	189.0	94.5	.06	7.6	.009
Residuals	18	222.4	12.4	.07		
Total	23	3002.8		1		

Browning had no effect on sterol composition (*F*_2,20_ = 0.90, *p* = .46), whereas the effect of shading bordered at significance (*F*_2,20_ = 2.90, *p* = .06) (Table 2, Figure S7). The interaction between the two treatments was non‐significant (*p* = .14) for sterols.

**TABLE 2:** Summary of PERMANOVA for the effects of browning, shading removal and their interaction on biofilm sterol composition

	*Df*	*SS*	*MS*	*R* ^*2*^	*F value*	*p*
Browning	2	70.6	35.3	.07	0.9	.464
Shading	1	113.3	113.3	.11	2.9	.055
Browning × Shading	2	136.1	68.0	.13	1.7	.140
Residuals	18	708.2	39.3	.69		
Total	23	1028.2		1		

### 3.3 Field survey along a natural DOC gradient

**FIGURE 4:**
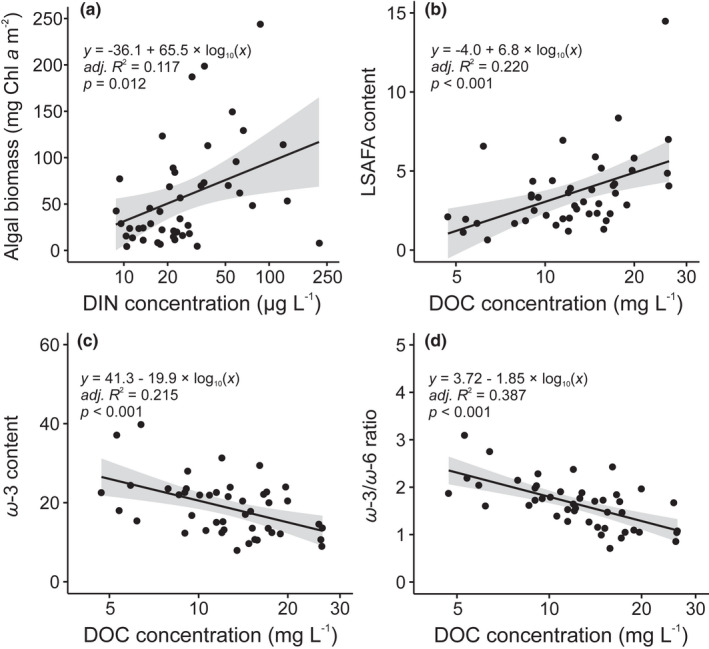
The relationships between (a) dissolved inorganic nitrogen (DIN) concentration and algal biomass (chlorophyll a) and dissolved organic carbon (DOC) and the content (%) of (b) long‐chain unsaturated fatty acids (LSAFA), (c) ω‐3 polyunsaturated fatty acids and (d) the ratio of ω‐3 and ω‐6 polyunsaturated fatty acids in biofilms of 45 Finnish headwater streams. The summary statistics of the least squared linear regression models are also depicted. The shaded area represents ±95% CI for the linear fit. The scales of the horizontal axes are log_10_‐transformed.

Algal biomass in the 45 study streams was controlled primarily by DIN concentration which, as the sole significant predictor, provided the lowest BIC value and explained 11.7% of algal biomass (Table 3; Figure 4a). In contrast, biofilm nutritional quality responded strongly to the DOC‐gradient. DOC was the key predictor of the proportion of LSAFA fatty acids (as % of all fatty acids; positive relationship) [Figure 4b] as well as of *ω‐*3 fatty acids and the *ω‐*3/*ω‐*6 ratio (negative relationships) [Table 3, Figure 4c and d]. EPA was related to a combination of DOC (negative relationship), pH, water temperature and DIN (all positive), which together explained 41.9% of variation in EPA content (Table 3).

**TABLE 3:** Summary of the most parsimonious linear regression models fitted to key environmental variables and algal biomass (mg Chl a m^−2^), contents (%) of LSAFA, eicosapentaenoic acid (EPA; 20:5ω‐3), ω‐3 polyunsaturated fatty acids and the ratio of ω‐3 and ω‐6 polyunsaturated fatty acids in the 45 field survey streams

Response variable	Predictors	Estimates	CI	*t*	*p*	Cum. Adj. *R* ^*2*^
Algal biomass	(Intercept)	−36.14	−110.35–38.08	−0.98	.332	
	DIN (log_10_)	65.47	14.92–116.02	2.61	.012	.117
L‐SAFA	(Intercept)	−3.03	−7.07–1.01	−1.51	.138	
	DOC (log_10_)	9.76	5.64–13.88	4.78	<.001	.220
	DIN (log_10_)	−3.05	−5.29 to −0.81	−2.74	.009	.322
EPA	(Intercept)	−59.97	−94.95 to −24.98	−3.46	<.001	
	DOC (log_10_)	−8.67	−16.16 to −1.18	−2.34	.024	.020
	pH	6.13	2.22–10.05	3.17	.003	.133
	Temperature (log_10_)	30.01	12.98–47.05	3.56	<.001	.330
	DIN (log_10_)	4.42	0.96–7.87	2.58	.014	.419
*ω*‐3	(Intercept)	41.32	28.64–54.00	6.57	<.001	
	DOC (log_10_)	−19.92	−31.03 to −8.81	−3.62	<.001	.215
*ω*‐3/*ω*‐6 ratio	(Intercept)	3.73	2.93–4.52	9.47	<.001	
	DOC (log_10_)	−1.85	−2.55 to −1.15	−5.36	<.001	.387

## 4 DISCUSSION

Browning of inland waters is continuing across the boreal region and can be expected to exert a strong control over primary production in all types of freshwater ecosystems. Nevertheless, almost all research on the ecological consequences of browning has focused on lakes, and most of the lentic studies are about pelagic productivity. Much less is known about how benthic algal production relates to browning, despite recent studies having showed that periphytic primary production may contribute greatly to whole‐ecosystem productivity in small humic lakes, even shifting these lakes towards net autotrophy (Vesterinen et al., 2016). In one of the few exceptions, Fork, Karlsson, and Sponseller (2020) demonstrated a unimodal response of benthic algal production along a gradient of DOC in northern Swedish lakes, much like that observed in lake pelagic zones (e.g. Bergström & Karlsson, 2019; Kelly et al., 2018). The unimodal pattern in pelagic primary productivity in lakes reflects a shift from nutrient‐limitation to light‐limitation, with a highly variable threshold from around five (Seekell et al., 2015) to 15 mg DOC L^−1^ (Bergström & Karlsson, 2019), and in some cases even up to 20 mg DOC L^−1^ (Olson et al., 2020). Fork, Karlsson, and Sponseller (2020) documented a corresponding shift at 8–9 mg DOC L^−1^ for primary production by lake periphyton. They further suggested that as most lakes in their study area in northern Sweden have currently very low DOC concentrations, their future benthic primary production will likely show mainly positive responses to increasing DOC. The ambient DOC level in our experiment was close to the peak value reported by Fork, Karlsson, and Sponseller (2020) and algal biomass gain was indeed highest in this treatment, decreasing substantially thereafter. Therefore, our moderate and even high DOC treatments should represent a realistic browning scenario for boreal headwater streams. Algal biomass response to reduced shading was much less dramatic than to browning, and to the opposite direction; as expected, removal of shading increased algal biomass accrual, but the effect was additive; that is, there was no interaction between browning and light augmentation.

Our field observations on benthic algal biomass deviated from those in the mesocosm experiment. Algal biomass in the field was primarily controlled by DIN while DOC (or any other environmental variable measured) was not an important factor. Several studies have shown phytoplankton in boreal lakes to be largely N‐limited (e.g. Bergström, 2010; Isles et al., 2020; Lau et al., 2021) and also a recent study in northern Swedish streams reported benthic algal biomass to be driven by an interaction between DIN availability and incident light levels while DOC had a minor role (Burrows et al., 2021). However, biofilm quality may be much more important than quantity to trophic transfer from primary producers to aquatic consumers, as has been previously observed for the phytoplankton‐zooplankton interaction in lakes (Gladyshev et al., 2010). Also several stream studies have suggested that the role of food nutritional quality to energy transfer in food webs is more important than that of food quantity, and that algal PUFA content is a reliable indicator of basal food nutritional quality (Guo, Kainz, Sheldon, & Bunn, 2016; Lau et al., 2009; Torres‐Ruiz et al., 2007).

Invertebrate consumers retain effectively algal long‐chain PUFAs which support predatory invertebrate and fish production in lakes (Lau et al., 2014) and probably even more so in streams where algal DHA is available only in low amounts (Guo et al., 2018). Furthermore, trophic support by algal long‐chain PUFAs extends across the water‐land interface, enhancing the breeding success of, for example, insectivorous birds feeding on emerging aquatic insects (Twining et al., 2016, 2019). In streams, diatoms have a prominent role in supporting higher trophic levels because other key components of stream periphyton, green algae and cyanobacteria, do not contain long‐chain PUFAs that are physiologically important for aquatic invertebrates (Hill et al., 2011; Torres‐Ruiz et al., 2007). The traditional view of terrestrial leaf litter and associated microbial decomposers, fungi and bacteria (which lack EPA and DHA) as the foundation of lotic food webs in forested landscapes has been challenged by studies showing that the nutritional quality of leaf litter per se is relatively low but greatly enhanced by the presence of algal biofilm on leaf surfaces (Guo, Kainz, Valdez, et al., 2016). Algae, even if available in very low amounts, may reduce the EPA limitation of lotic consumers and therefore have a primary role in supporting secondary production (Crenier et al., 2017; Guo et al., 2021).

The highest browning level in our experiment reduced benthic algal biomass from that in the ambient control by about 80%. Biofilm quality (as indicated by EPA, *ω‐*3 fatty acid and sterol content) collapsed even more, and almost the same level of reduction was caused already by a relatively slight increase in DOC concentration from the average ambient of 9.7 to 13.2 mg L^−1^. With increasing browning, biofilm fatty acid composition became dominated by long‐chain saturated fatty acids (LSAFA), that are characteristic fatty acids for tPOM. Previous study with boreal lakes revealed higher allochthony of cladocerans in brown‐water lakes than in lakes with lower DOC concentrations due to the suppressed availability of phytoplankton (Taipale, Vuorio, et al., 2016). Several studies have indicated that although aquatic consumers may ingest and assimilate t‐POM and associated bacteria, this is mainly because of a limited availability of better dietary sources, and allochthonous organic matter provides only weak support to growth and reproduction of aquatic consumers, for example *Daphnia* (Hiltunen et al., 2019; McMeans et al., 2015; Taipale et al., 2015) and isopod *Asellus aquaticus* (Lau et al., 2014). Our experimental water browning induced a dramatic decrease in biofilm quality not only in terms of impaired fatty acid composition but also the total sterol content of the biofilm decreased sharply with increased browning. This is an important observation because arthropods are generally incapable of synthesizing sterols but need to obtain them from their diet (Goad, 1978). Invertebrates can, however, use algal phytosterols as precursors of their main type of sterol, cholesterol (Martin‐Creuzburg & von Elert, 2004). Such an impairment of biofilm quality, the major high‐quality food source available for stream invertebrates, will likely have far‐reaching consequences to energy transfer in stream food webs, particularly as the quantity of periphytic algae decreased parallelly.

Our key finding from the mesocosm experiment was that EPA, a physiologically important fatty acid for stream consumers, was greatly reduced by browning and less so by canopy shading. The fatty acid profiles of algae are largely phylogenetically conserved (Dalsgaard et al., 2003; Taipale et al., 2013) and periphyton fatty acid composition therefore reflects the taxonomic composition of algal assemblages (Honeyfield & Maloney, 2015). Although we did not directly quantify algal community composition via microscopy or any other means, changes in EPA very likely reflect the relative abundance of diatoms in our samples. In the field data, another frequently used indicator of resource quality, the ω3/ω6 ratio, responded strongly negatively to increasing DOC concentrations whereas EPA was not as clearly related to any particular environmental factor but was rather controlled by a complex set of variables, most of which (nutrients, DOC, pH) are associated with water browning. We also did not observe any reduction of periphyton PUFA content in our non‐shaded treatments; by contrast, PUFA content was higher in all non‐shaded treatments, closely tracking the increased abundance of diatoms with light augmentation. Nonetheless, the difference to most previous studies documenting fatty acid responses to light may be more apparent than real. For example, Hill et al. (2011) showed that while light increased saturated and *ω‐*6 fatty acid content of benthic biofilm, *ω‐*3 fatty acids remained constant or decreased slightly (see also Huggins et al., 2004).

Using a large body of published literature on fatty acid profiles of marine and freshwater phytoplankton, Hixson and Arts (2016) showed that increased temperature was strongly correlated with a decrease in the proportion of *ω‐*3 PUFAs and increase in *ω‐*6 PUFAs and saturated FAs. They further predicted a global 8.2% reduction in EPA production with a 2.5 °C increase in water temperature. Considering the key role of EPA and other LC‐PUFA to vital organismal functions, such reduction could have cascading effects on both aquatic and terrestrial food webs. We detected a corresponding but even stronger reduction of *ω*‐3 fatty acids in stream periphyton induced by water browning. Our data suggested that the abundance of diatoms decreased drastically, which was then reflected as a greatly reduced availability of LC‐PUFAs already with a moderate increase of DOC concentration. As even the highest DOC levels in our experimental design are not uncommon today, our experiment should represent a very realistic browning scenario for boreal headwater streams, potentially resulting in a major shift in trophic support from algal primary production to allochthonous DOM (Karlsson et al., 2015), with an associated reduction in trophic transfer efficiency to stream and riparian consumers.

## CONFLICT OF INTEREST

The authors declare no competing interests.

## Supporting information


Appendix S1

## Data Availability

The data that support the findings of this research are openly available in Dryad at https://doi.org/10.5061/dryad.9w0vt4bj3.
