# Supplementary material for: Dark matters: Contrasting responses of stream biofilm to browning and loss of riparian shading
Source: Glob Chang Biol. 2022 Jun 15;28(17):5159–71. doi: 10.1111/gcb.16279 (PMC9545655; doi:10.1111/gcb.16279)

**Supporting information: Dark matters – contrasting responses of stream biofilm to browning and loss of riparian shading**

Jussi Jyväsjärvi^1^, Maria Rajakallio^1^, Joanna Brüsecke^1^, Kaisa-Leena Huttunen^1^, Ari Huusko^2^, Timo Muotka^1^, Sami J. Taipale^3^

^1^Ecology and Genetics Research Unit, University of Oulu, P.O. Box 3000, FI-90014, Finland

^2^Natural Resources Institute Finland (Luke), Paltamo, Finland

^3^Department of Biological and Environmental Science, University of Jyväskylä, Finland

* Correspondence to: Jussi.jyvasjarvi@oulu.fi

**Table S1**. Averages and minimum and maximum values of the key environmental variables in the 45 field survey streams.

|  |  |  |  |
| --- | --- | --- | --- |
| **Variable** | **Mean** | **Min** | **Max** |
| Catchment area (km^2^) | 7.5 | 0.2 | 25.8 |
| Mean stream width (m) | 2.0 | 0.5 | 5.4 |
| Mean stream depth (cm)* | 19.0 | 9.5 | 32.3 |
| Current velocity (cm s^-1^)* | 22.2 | 4.5 | 39.6 |
| Canopy cover (%)*^†^ | 37.6 | 16.4 | 53.7 |
| Mean temperature (°C; Aug-Sep)* | 9.9 | 7.0 | 13.2 |
| pH* | 6.6 | 5.9 | 7.3 |
| Phosphate phosphorus (PO_4_; µg L^-1^)* | 5.5 | 0.2 | 28.4 |
| Dissolved inorganic nitrogen (DIN; µg L^-1^)* | 36.4 | 8.8 | 220.0 |
| Dissolved organic carbon (DOC; mg L^-1^)* | 13.5 | 4.7 | 26.2 |
| * Variable included in the all-subsets model selection |  |  |  |
| ^†^ Measured with GLAMA mobile application |  |  |  |

**Table S2**. Lists of individual fatty acids used for the characterization of specific fatty acid groups.

| **ω-3** | **ω-6** | **LSAFA** | **BFA** |
| --- | --- | --- | --- |
| 16:4ω3 | 16:1ω6 | 22:0 | i-14:0 |
| 16:4ω1 | 16:2ω4 | 24:0 | i-15:0 |
| 18:3ω3 | 18:2ω6 | 25:0 | a-15:0 |
| 18:4ω4 | 18:3ω6 | 26:0 | i-16:0 |
| 18:4ω3 | 20:2ω6 | 27:0 | a-17:0 |
| 20:3ω3 | 20:3ω6 | 28:0 | 17:0 |
| 20:4ω3 | 20:4ω6 | 29:0 |  |
| 20:5ω3 | 22:4ω6 | 30:0 |  |
| 22:5ω3 | 22:5ω6 |  |  |
| 22:6ω3 |  |  |  |

**Figure S1**. Aerial image of the experimental arena at the Natural Resources Institute Finland’s research station (Kainuu Fisheries Research Station (N 64° 24.240’ E 27° 31.320’)), Paltamo, central Finland. The locations of water inflow to the system, the upstream section, head tanks for the humin feed supply and the experimental flumes are delineated by dashed lines.


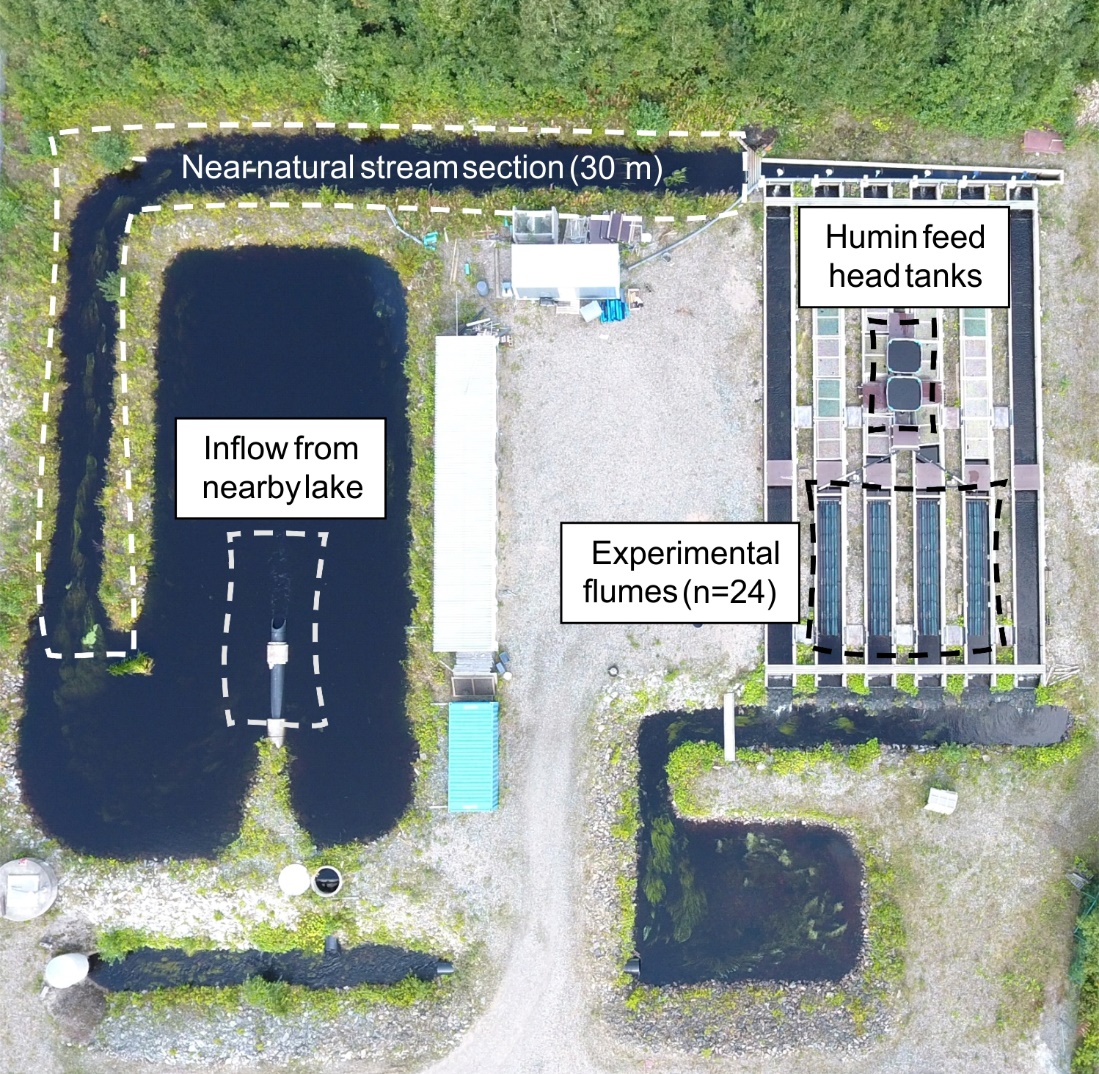


**Figure S2**. Daily average a) light intensity (1000 lux; 10 am – 4 pm) and b) water temperature (°C) in different treatment combinations during the experiment.


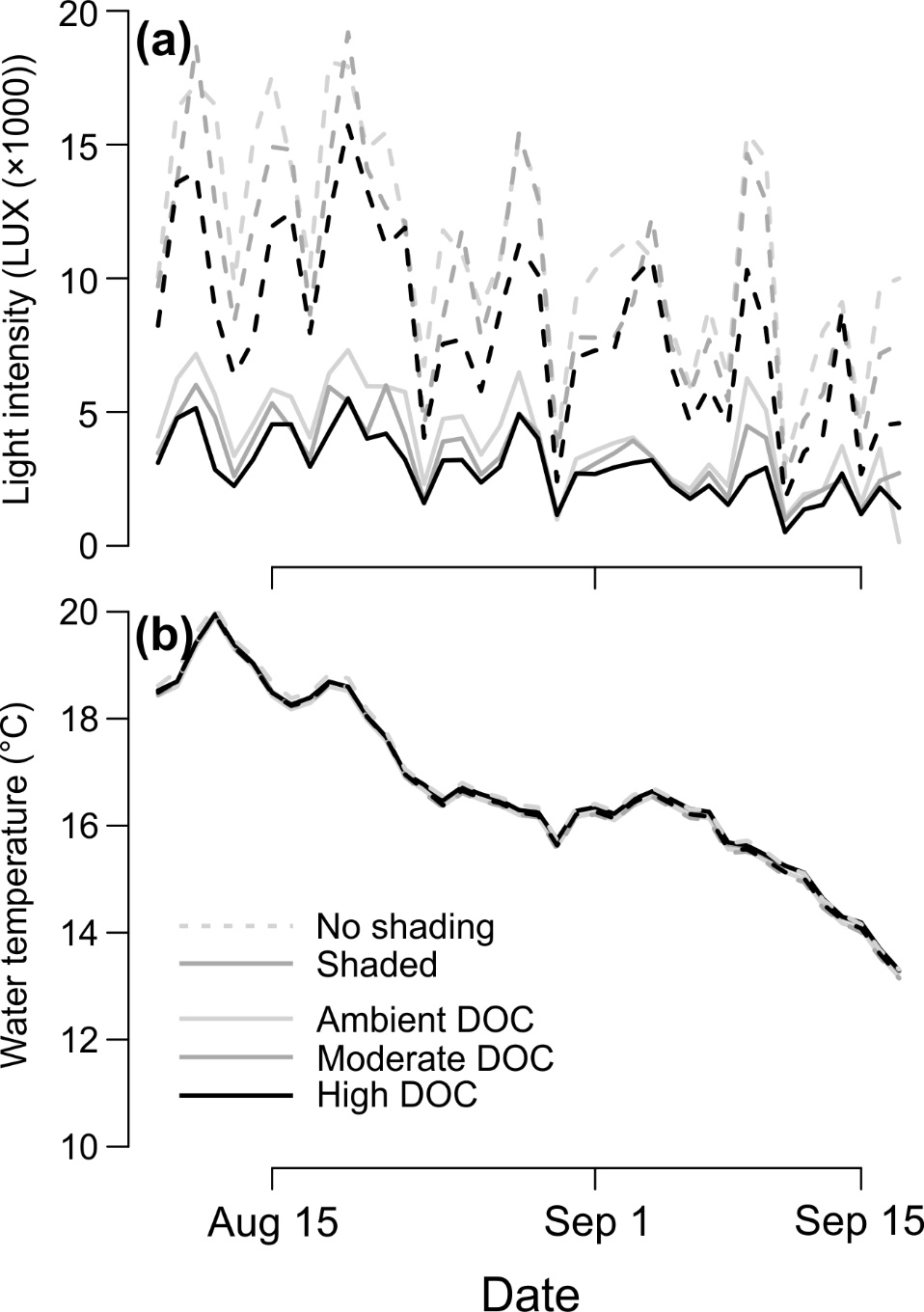


**Figure S3**. Variation of water absorbance (436 nm) values (mean ± SE) in the three browning treatments during the experiment. Dashed thick horizontal lines represent average absorbance values of the three browning treatments during the entire experiment.

**
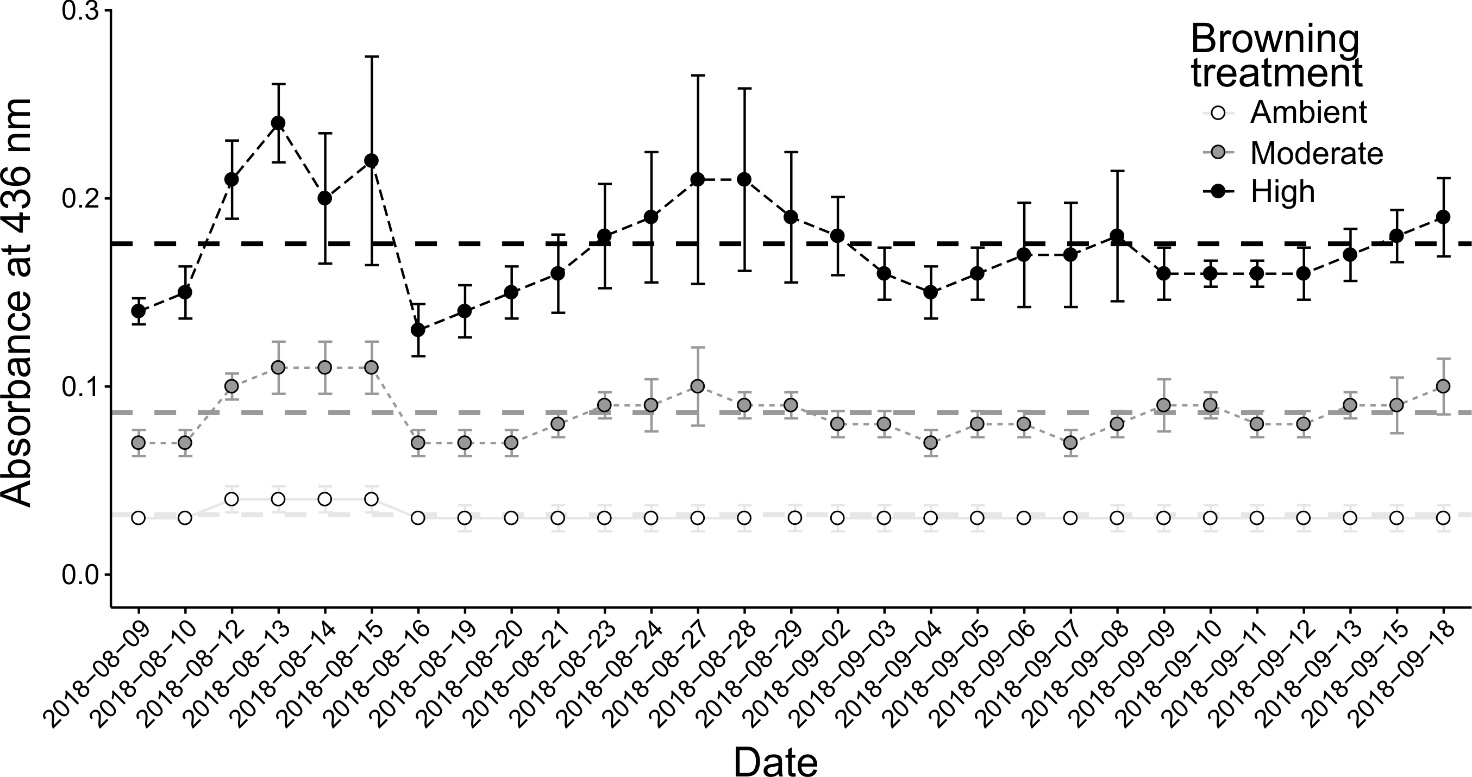
**

**Figure S4**. Distribution of 4581 water colour (mg Pt L^-1^) records from Finnish streams during 2010-2017 (data source: HERTTA-database of the Finnish Environment Institute). Horizontal lines indicate the three experimental browning treatments (solid lines) and 20%, 75% and 98% quantiles of the distribution of national water colour observations (dashed lines).

**
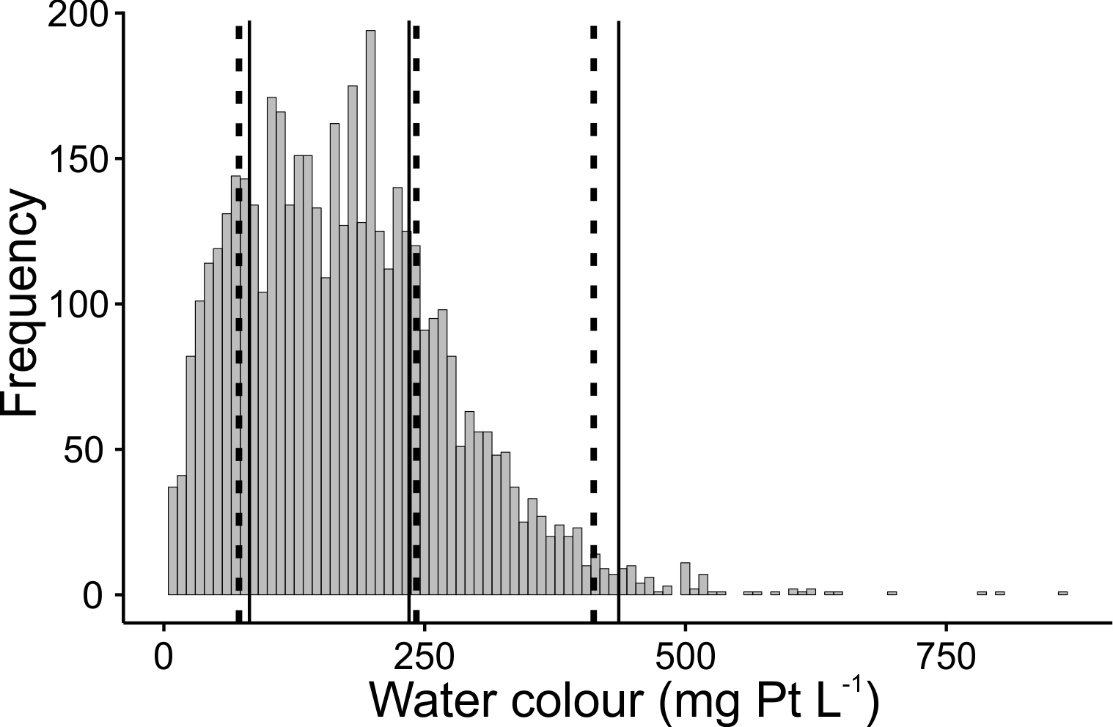
**

**Figure S5**. Locations of the 45 field survey streams in central Finland. The colours of the circles represent the dissolved organic carbon (DOC, mg L^-1^) concentration of the stream water.

**
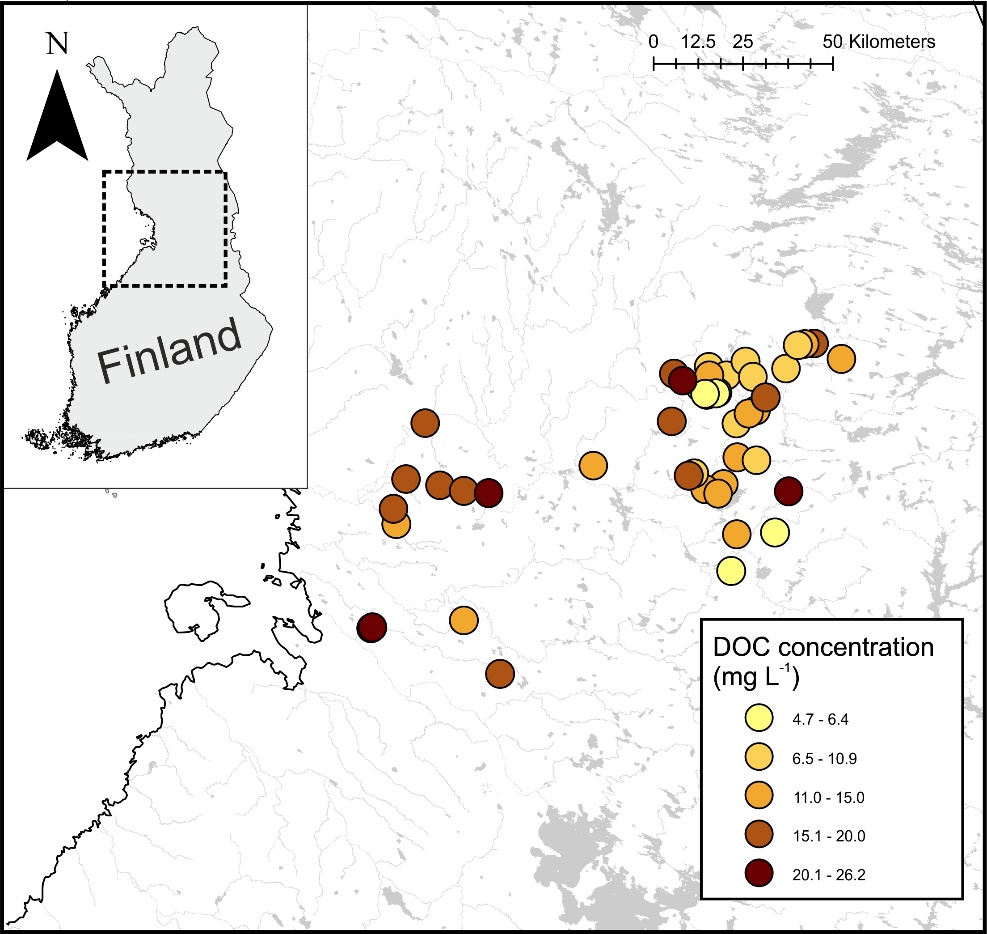
**

**Figure S6**. Mean (± 95% CI) a) ω-6 polyunsaturated fatty acid and b) bacterial fatty acid (BFA) content in each experimental treatment

**
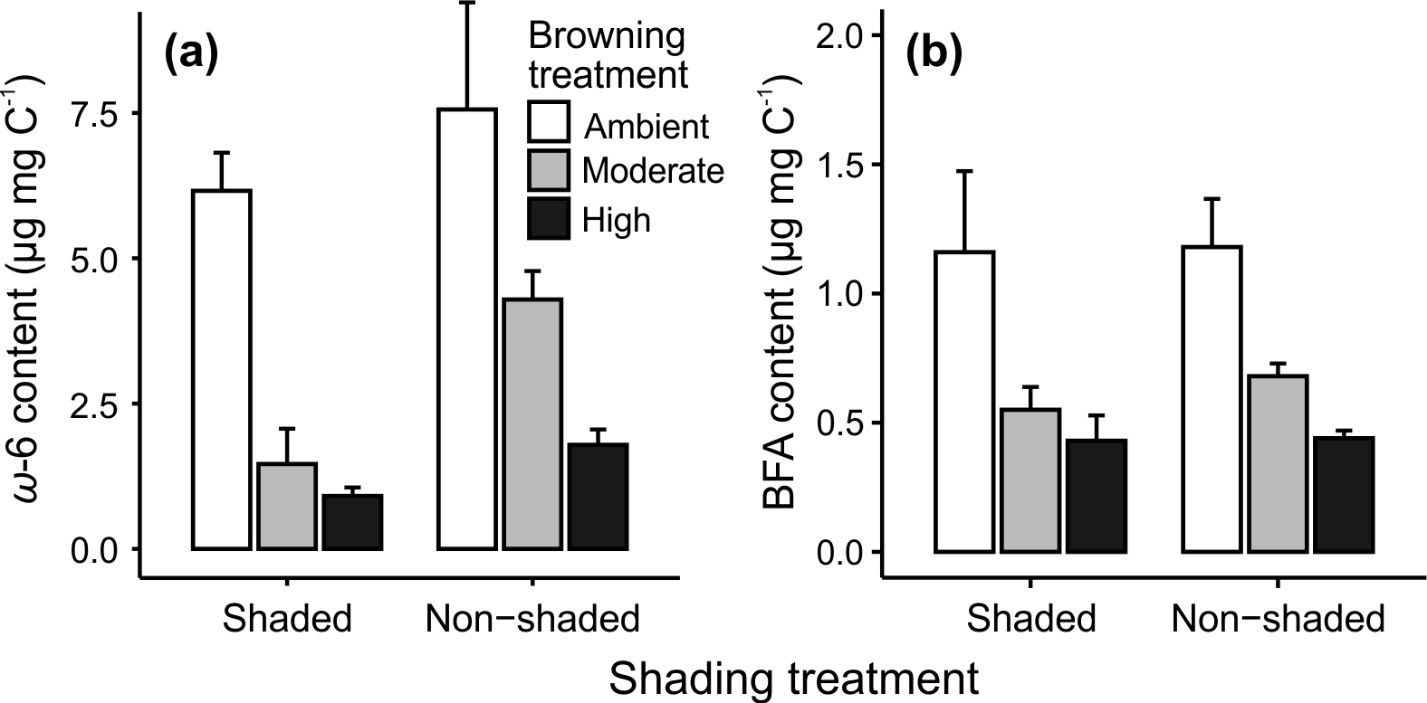
**

**Figure S7**. NMDS ordination of the sterol composition, showing separation of the experimental treatments in the ordination space. Light-grey ellipses in panel a depict 95% confidence interval ellipses around the treatment centroids.


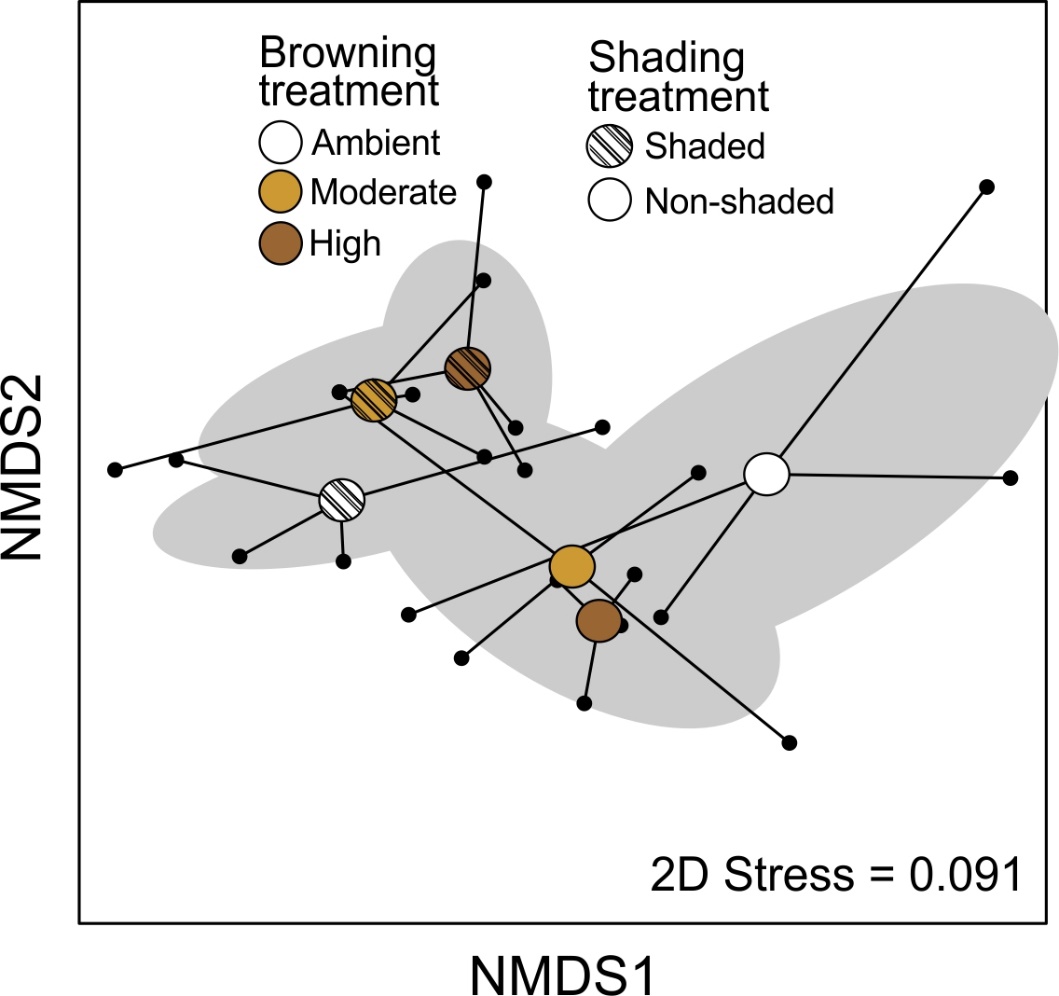

Supplement: Supplementary file 1 — Appendix S1 [file GCB-28-5159-s001.docx]
